# Striking a Balance: Innovation, Equity, and Consistency in AI Health Technologies

**DOI:** 10.2196/57421

**Published:** 2025-04-07

**Authors:** Eric Perakslis, Kimberly Nolen, Ethan Fricklas, Tracy Tubb

**Affiliations:** 1 Duke Clinical Research Institute, Duke University School of Medicine, Durham, NC, United States; 2 Pluto Health, Durham, NC, United States; 3 Pfizer Inc, New York, NY, United States

**Keywords:** artificial intelligence, algorithm, regulatory landscape, predictive model, predictive analytics, predictive system, practical model, machine learning, large language model, natural language processing, deep learning, digital health, regulatory, health technology

## Abstract

With the explosion of innovation driven by generative and traditional artificial intelligence (AI), comes the necessity to understand and regulate products that often defy current regulatory classification. Tradition, and lack of regulatory expediency, imposes the notion of force-fitting novel innovations into pre-existing product classifications or into the essentially unregulated domains of wellness or consumer electronics. Further, regulatory requirements, levels of risk tolerance, and capabilities vary greatly across the spectrum of technology innovators. For example, currently unregulated information and consumer electronic suppliers set their own editorial and communication standards without extensive federal regulation. However, industries like biopharma companies are held to a higher standard in the same space, given current direct-to-consumer regulations like the Sunshine Act (also known as Open Payments), the federal Anti-Kickback Statute, the federal False Claims Act, and others. Clear and well-defined regulations not only reduce ambiguity but facilitate scale, showcasing the importance of regulatory clarity in fostering innovation and growth. To avoid highly regulated industries like health care and biopharma from being discouraged from developing AI to improve patient care, there is a need for a specialized framework to establish regulatory evidence for AI-based medical solutions. In this paper, we review the current regulatory environment considering current innovations but also pre-existing legal and regulatory responsibilities of the biopharma industry and propose a novel, hybridized approach for the assessment of novel AI-based patient solutions. Further, we will elaborate the proposed concepts via case studies. This paper explores the challenges posed by the current regulatory environment, emphasizing the need for a specialized framework for AI medical devices. By reviewing existing regulations and proposing a hybridized approach, we aim to ensure that the potential of AI in biopharmaceutical innovation is not hindered by uneven regulatory landscapes.

## Introduction

### Background

The convergence of algorithms, artificial intelligence (AI), big data, and digital health technologies (DHTs) is a sea change not seen since the “dot.com” era, which has significantly changed the way we work, play, and learn [1,2]. However, the lack of comprehensive regulatory guidance has led to the force-fitting of novel innovations into existing categories, leading to ambiguous boundaries between medical devices and consumer electronics. This results in added ambiguity for innovators seeking to share valuable product concepts. What is lacking is a comprehensive approach to evaluating medical benefits, risks, and evidence that can be universally applied across different product categories, product types, and regulatory regimes [3]. Such an approach would be flexible, allowing for the distinctions between various products to be properly addressed. Clear regulations play an important role in enabling easier scaling, highlighting the mutually beneficial relationship between regulatory clarity and the acceleration of innovation. Moreover, the pharmaceutical industry’s comprehensive understanding of scaling and marketing extends beyond the confines of drug development, presenting a valuable paradigm for other sectors in the AI landscape. This paper addresses the ambiguity faced by innovators and proposes models for evidence strategies, particularly focusing on the distinct regulatory challenges faced by the biopharma industry.

At the time of this writing, Gartner [4] has placed the increasingly popular generative AI technology at the peak of inflated expectations for emerging technologies in 2024. Whether the transformation that generative or other forms of AI and DHTs bring to health care occurs gradually or rapidly, there is widespread anticipation of both advancement and potential challenges [5,6]. The many use cases ranging across diagnostics, logistics, clerical improvements, and new treatment modalities in general medicine and across medical subspecialties have been described in detail within the literature [7,8]. Similarly, the use of these technologies holds equally compelling promise within biomedical product development [9,10].

### Current Challenges

AI and DHTs represent a wide range of intricate and interconnected technologies, and the vast array of applications are equally diverse and complex. For example, most DHTs rely on proprietary algorithms trained from and across a mixture of private and public data sources. As multiple technologies are integrated into a tool, more information may be shared, and the capabilities and risks aggregate [11]. This additive complexity challenges traditional domain-based regulatory regimes. The US Food and Drug Administration (FDA) regulates medical devices, but not all algorithms and apps meet the regulatory definition of a medical device as defined in Section 201(h) of the Food, Drug, and Cosmetic Act. These apps often access and transmit data across the internet but do not fit neatly into the codified remit of the Federal Communications Commission or the Federal Trade Commission (FTC) [12]. Even within regulatory regimes, there are qualified gaps. The Office for Civil Rights enforces health care privacy, but only for covered entities, leaving a loophole and resulting in gaps in protection that are systematically being exploited [13]. Further, these overlapping, complex, and intricate interregulatory and intraregulatory regimes create confusion and inequities, hindering progress.

### Objectives

While there have been efforts to establish standardized approaches to the regulatory assessment of DHT and AI medical products, many of these frameworks take the approach of a single regulator and regulatory regime versus approaches that inform regulatory decision-making across the spectrum of relevant regulators [14-16]. Further, these frameworks incorrectly assume that all innovators are alike. Consumer electronic companies and health technology startups, providing solutions that may overlap or compete with offerings from traditional medical device and pharmaceutical companies, often navigate a regulatory landscape that offers them more adaptability in their operations, which differs from the established health care regulations governing other sectors. The coexistence of unregulated and highly regulated makers in the same market can lead to various challenges, including issues related to safety, quality, and fair competition. Balancing innovation and oversight is crucial in this context. We need solutions that promote fair competition while maintaining a high standard of safety and product effectiveness, without creating a disparity between the heavily and lightly regulated entities. It is also helpful to level set on terminology. Textbox 1 provides definitions for common terms used in this space.

Textbox 1. **Key terms and definitions.**

Digital health technologies: technologies consisting of hardware (eg, sensors or transmitters) or software (eg, connectivity software, algorithms, or artificial intelligence) components that are used for health care–related purposes.

Medical device software: term primarily used in the European Union to define software with a medical purpose that can be used either alone or in combination with a regulated medical device. This is not interchangeable with software as a medical device [17].

Software as a medical device: software that is used for medical purposes and may do so independently of a hardware medical device as well as not being a required component of a hardware medical device [18].

Mobile medical apps: mobile apps serving as medical devices, which integrate software functionality that aligns with the Food and Drug Administration definition of a device, as outlined in Section 201(h) of the Food, Drug, and Cosmetic Act. These apps may function as accessories to regulated medical devices or convert a mobile platform into a regulated medical device [19].

Digital therapeutics: software-based interventions intended to prevent, manage, or treat medical conditions based on evidence of a demonstrable positive therapeutic impact on a patient’s health [20].

Direct to consumer: marketing products or services directly to consumers without the involvement of a health care provider.

## Regulatory Regimes and Industries in DHT

The key is that not all makers are subject to regulations in the same manner nor do they exhibit the same affinity for risk. For example, while direct-to-consumer advertising is highly regulated for pharmaceutical products, the oversight is less consistent for over-the-counter (OTC) “devices,” such as some medical tests not regulated by the FDA or FTC [21,22]. This not only results in a less than comprehensive regulatory coverage of AI medical devices and DHTs but also involuntarily creates an ecosystem where makers develop and market their products around the varying gaps in regulatory coverage. Certain products, such as OTC medical device algorithms to detect sleep apnea, may be subject to less regulation and thus have an advantage over established health care products. OTC sleep apnea devices represent a category of products that can fall in the “interstitial spaces” of regulatory oversight, as they are not always subject to the same level of scrutiny as prescription devices. These products often include wearable sensors, smartphone apps, or other consumer-grade devices that purport to detect symptoms of sleep apnea, such as disrupted breathing or low oxygen levels during sleep. Many OTC sleep apnea devices may fall into class I or II and thus may not require premarket approval, which is the most stringent type of device marketing application required by the FDA. Instead, they may only need to meet the requirements for 510(k) clearance, which is a less rigorous process and does not require clinical trials. However, there are also some OTC sleep apnea devices that do not fall under any FDA regulation because they are marketed as “wellness” or “lifestyle” products rather than medical devices. They do not detect signs of sleep apnea and are not marketed as a medical device but as a sleep improvement system; therefore, they do not fall under FDA regulation.

This lack of consistent regulation can create opportunities for companies to market products with less oversight and potentially greater profit margins, but it can also lead to consumer confusion and potential safety risks if the products do not perform as advertised. It is a clear example of how the existing regulatory framework may struggle to keep up with the rapid pace of innovation in DHT. Clear and well-defined regulations play a pivotal role, especially during the transition from exploratory phases to scaling products, enabling smoother, efficient scaling processes.

In many ways, the opposite situation exists for larger established health care organizations. A highly regulated pharmaceutical company that is already subject to the many previously discussed compliance regimes and other complex corporate regulatory obligations may find it too difficult or risky to attempt digital innovation, as the burden of reporting, evidence, and oversight are all greatly heightened compared to niche innovators. This scenario must be discouraged, as these organizations have deep expertise within the disease areas where they have successfully delivered drugs and devices. This expertise could lend itself to success beyond many health technology startups, which often fail due to a lack of market fit of their products [23]. Encouraging a balance between regulatory clarity and flexibility is paramount to fostering innovation across diverse players in the digital health landscape. Indeed, there is a cost to regulatory compliance, which is more readily absorbed by well-resourced companies. Smaller startups may not have sufficient funding to run the optimal size and scale validation study. They may have funding constrained by the need to showing promise to investors in order to survive to their next round of funding.

## Evidence Requirements and Claims

While one side of the matrix is the nature of the products being developed and the types of makers, the evidence supporting these products is equally if not more diverse. Companies with very different sizes and areas of expertise may be competing openly within a range of product categories and evidence strategies with clinical development plans that seem lacking. Much of this may be attributed to the relative lack of maturation of the AI medical device and DHT spaces, which has led to a wide range of interpretation of the guidelines. This can pose challenges for companies with more rigid regulatory boundaries, which wish to participate in this evolving experimental domain and have substantial evidence strategies to support product development but are uncomfortable as the space is not mature. For most, the first step is to determine the type of product being developed. However, when dealing with products designed for medical purposes or functions, it is essential to ensure that it addresses an unmet medical need. In the United States, the product type can vary from a device software or algorithm that may be classified as mobile medical apps, software functions that are not medical devices, clinical decision support software, or software as a medical device (SaMD) [24-26]. Each of these product types needs different types and levels of evidence to support them in the market and may need regulatory approval. While the FDA offers guidance on how to determine the product type, significant judgment is required due to similarities within the categories as well as the severity of the disease indication. This necessitates an iterative thought process, considering multiple regulatory guidance alongside the evidence strategy and clinical development plan [27].

**Figure 1.**
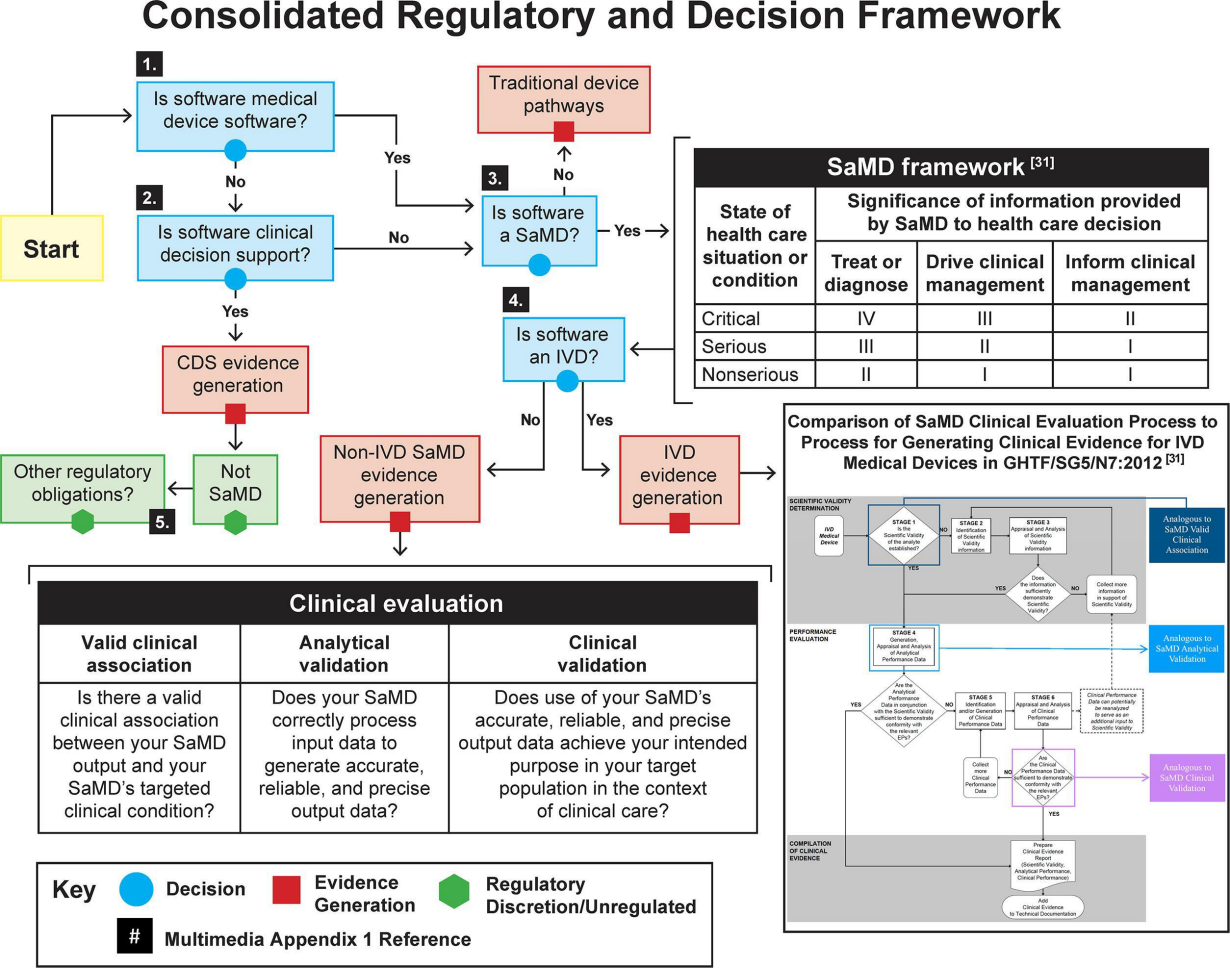
Consolidated regulatory classification decision framework (for the reader’s convenience, Multimedia Appendix 1 gives a set of figures referenced in Figure 1). CDS: clinical decision support; IVD: in vitro diagnostic; SaMD: software as a medical device.

To simplify this process, we developed the graphical consolidated regulatory decision framework shown in Figure 1 [28-30]. This framework builds on the approach in the FDA Guidance, Software as a Medical Device (SAMD): Clinical Evaluation [31]. Additional details supporting this framework are available in Multimedia Appendix 1. A precursor to the workflow is determining whether the clinical association is well-established or novel. This can be nontrivial, as many SaMD products lack clinically established standards due to the novelty of the product. When there is a well-established clinical association, these SaMD have outputs with well-documented association as identified in sources such as clinical guidelines, clinical studies in peer-reviewed journals, consensus for the use of the SaMD, international reference materials, or other similar well-established comparators of previously marketed devices. When the clinical association is novel, these SaMD may involve new inputs, algorithms, outputs, new intended target population, or new intended use. An example may include the combination of nonstandard inputs (eg, mood or pollen count), with standard inputs (eg, blood pressure or other physiological signals), that uses novel algorithms to detect deterioration of health or diagnosis of a disease.

The importance of objective consideration of these questions cannot be overstated. Frequently, innovators have embedded biases within their assumptions that cloud judgment in this assessment.

Any one or combination of these biases can threaten or derail the development of novel technology products including product integrity and patient safety. For example, the well-publicized case of the FDA warning letter that caused Owlet to cease selling their Smart Sock and copackaged products includes several of these biases [32]. The FDA determined that the product was a medical device but the maker had not reached the same determination [33]. According to the FDA, the apnea alarms had inadequate clinical evidence, and parents could potentially seek emergency care due to product alarms that had inadequately established clinical association (prestep), specifically dips in oxygen saturation as determined by pulse oximetry in infants during sleep [34]. This incident exemplifies how the misclassification of a product type and inadequate clinical evidence highlight the challenges in navigating ambiguous regulatory guidelines. Clear regulations not only mitigate the risk of inadequate clinical evidence, reducing the likelihood of triggering unnecessary care in this case, but also highlight the significance of aligning evidence strategies with robust clinical development plans to avoid such pitfalls.

In contrast, Apple’s approach to irregular heartbeat notification serves as an example that a technology company that operates outside the traditional health care sphere can diligently address product-type classification and evidence. Apple developed these FDA-cleared features via rigorous and traditional approaches using randomized controlled trials [35,36]. The resulting product label is considered FDA-regulated; therefore, the claims made about the product must not exceed the evidence produced, the specifics listed within the clearance letter, or the resulting label [37]. However, Apple is an exception within the technology industry in size, scope, and resourcing. The average medical device maker is much smaller and must build their product strategies around regulatory regimes in order to get their product to market. This is not solely about the avoidance of regulation. Many innovations pass the bar for regulatory clearance but not the bar for reimbursement, which can be a difficult and unprofitable market situation. Alternatively, developing a product that does not meet the bar for FDA regulation can result in a highly profitable “health” or “fitness” application that, while not regulated or reimbursed by health insurance, can be highly profitable by volume of sales even at very low-price points.

If the maker determines that the software product is not a medical device, the next step is to determine whether the product is a decision support tool, and this can be accomplished with the aid of the earlier-referenced FDA guidance document.

Continuing with the framework, if the software is a medical device, the next step is to determine whether the software is a SaMD. If not, the user is directed toward traditional medical device pathways. If so, they are guided through the SaMD categorization process. This is complicated by the requirement for a deep understanding of the medical indication and all possible outcomes from any chosen route. Determining whether a SaMD treats or diagnoses, whether it drives clinical management, or simply informs clinical care yields a level of interpretation that often varies by stakeholders. In addition, it must be simultaneously determined whether these actions occur in a critical, serious, or nonserious medical situation. The FDA has published guidance on when and whether independent review of these decisions should be included.

Once the SaMD is categorized, the next step is to determine whether the product is an in vitro diagnostic (IVD) or non-IVD SaMD. The rubric directs the user toward evidence generation in either case. When the SaMD is non-IVD and novel clinically, the evidence generation process requires the establishment of a valid clinical association, analytical validation, and clinical validation of the product. These steps have been elaborated in detail within the previously referenced work by Goldsack et al [16]. When the SaMD is an IVD, the evidence generation process is analogous to the non-IVD case and can follow the stages of the clinical evidence assessment process as outlined in the Global Harmonization Task Force’s Clinical Evidence for IVD Medical Devices [38].

## Product Labeling Standards as a Guide

One improbable solution to creating clear and concise regulations would be the reorganization of current regulatory regimes to produce a new agency focused directly on the regulation of health care technologies. However, we can gain some insights from the nutrition industry.

Today, there are 3 agencies responsible for the regulation of food and nutrition information, the FDA, the FTC, and the Food Safety and Inspection Service (FSIS) of the US Department of Agriculture [39]. This may appear logical, assuming that each agency shares part of an overall mission. However, the reality is that handoffs, overlapping, and gray areas decrease the regulatory effectiveness or, at minimum, create confusion of roles and responsibilities. Continuing the example of food labeling, the FTC regulates food advertising, while the other 2 agencies share responsibility for regulating labels: FSIS regulates meat, poultry, and egg labeling and FDA regulates labeling for all other foods and nonspecified red meat (game). The Nutrition Labeling and Education Act addressed FDA-regulated packages and FSIS-issued parallel regulations. As an example of a gap, there are no provisions in the regulatory authorities defined by Congress that allow the FDA to approve dietary supplements for safety before they reach the consumer [40]. This results in fragmented safety data and little ability to forecast or prevent harmful products from reaching consumers [41].

**Figure 2.**
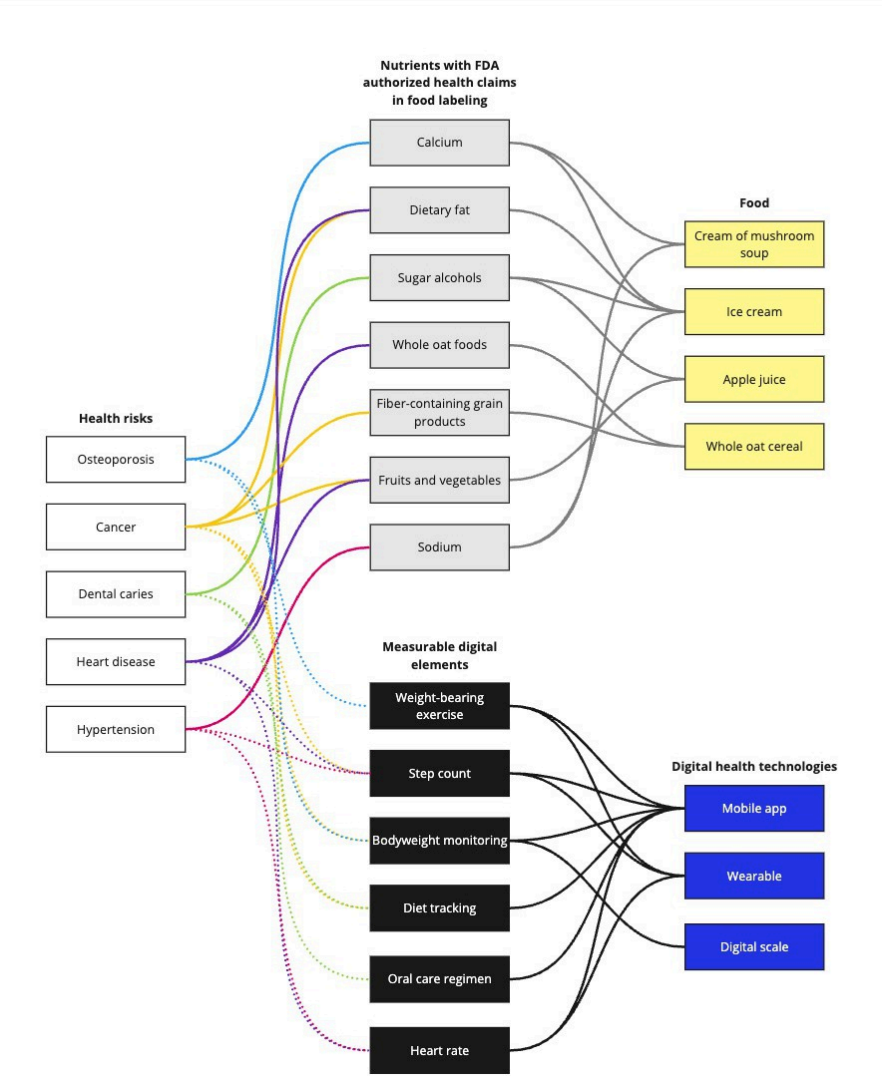
Health impacts and their associated nutrients and digital elements. FDA: Food and Drug Administration.

There is a great deal that the digital health space can learn from nutrition and food labeling. Specifically, the FDA or Nutrition Labeling and Education Act and the FSIS oversee 3 elements of food package labeling: nutrient content, nutrient content claims, and “disease” claims. Further, the FDA has restricted health claims to a small number of permitted claims [42]. This type of strategic and comprehensive approach to labeling is a model that if applied to AI medical devices and DHTs would improve transparency and clarify their benefits and risks. Elements are starting to appear in relevant subdomains of digital health such as cybersecurity, computing hardware, clinical decision support, and medical devices [43-45]. Examples extending the nutrition-based health claims into the digital domain are shown in Figure 2. This figure shows health risk areas linked to nutrients having FDA-approved health claims. The figure shows those same health risk areas linked to digital health elements that could also have an impact on risk. Standardized AI medical device and DHT product labeling across prescription and nonprescription products could address the diversity of innovators and makers just as nutrition labeling levels the field between small farms and large, industrial food production corporations, as all are held to the same standards.

Different aspects of the creation, oversight, and enforcement of such labeling regulations would likely fall within the purview of existing regulatory bodies assessing medical devices and algorithm-driven DHTs. As Table 1 suggests, this is a current patchwork of different agencies without necessarily one central authority. The table indicates how the different agencies could each play a role in the oversight of AI medical devices and DHTs and the relevant product development.

**Table 1.** Regulatory authority across components of medical devices and algorithm-driven digital health technologies (DHTs).

Federal agency	Relevant aspects of current role	Possible enhanced role
US Food and Drug Administration	Regulation of medical devices, MMAs^a^, SaMDs^b^	Develop specific guidelines for labeling AI^c^ medical devices, including continuous learning algorithms, and establish a clear pathway for marketing authorizations and DTC^d^
Federal Trade Commission	Enforcement of federal laws that prevent fraud, deception, and unfair business practices (as in advertising)	Regulate use of labels in DTC advertising of AI medical devices and DHTs
Centers for Medicare & Medicaid Services	Administers the Open Payments program, enhancing transparency by collecting and publicly disclosing information about financial relationships between health care providers and pharmaceutical or medical device manufacturers	Outline required transparency elements in the label, capturing health care provider financial interactions with companies in the design, testing, and use of DHTs
US Department of Health and Human Services, Office for Civil Rights	Ensures the privacy and security of protected health information	Ensure that the AI medical device label provides transparency in how AI devices use patient data and enforce penalties for noncompliance
National Institute of Standards and Technology	Develops and maintains technical standards	Establish the benchmarks cited on the label for AI performance, safety, and interoperability with other medical systems
US Consumer Product Safety Commission	Protects the public from the risk of injury or death associated with consumer products	Issue product recalls for all other DHTs or AI-enabled health care devices not under the Food and Drug Administration’s purview

a MMA: mobile medical app.

b SaMD: software as a medical device.

c AI: artificial intelligence.

d DTC: direct to consumer.

## Al-Based DHT Package Labeling

Regardless of the type of maker, a significant unmet need that can inform innovators’ strategies is standardized package labeling. Based upon the known potential harms and known potential limitations in AI-based DHTs, the minimum content for analogous product labeling would include the type of algorithm, the framework used for evidence generation, qualification and quantification of reproducibility, the ethical framework used, a description of the data used to train the model including how it was collected and consented, a statement on how bias was minimized or quantified, the risk management framework used to aggregate these various elements, and performance metrics [46-52]. This would enable a DHT packaging label, similar to the example shown in Table 2.

**Table 2.** Example of a permitted claims approach to artificial intelligence (AI)–based digital health technologies (DHTs).

DHT or algorithm design element	Permitted health claim	Example
Type of evidence basis	Primary benefit or utility	Randomized controlled trials, real-world evidence, etc
Ethical framework	Population benefit-risk	Inclusion and exclusion criteria as well as interpretability and explainability
Reproducibility	Statistically quantified and qualified claims	Specific indications and efficacy, data lineage, model versioning
Training data description	Applicability and specificity to populations	Rationale for included and excluded populations, training and testing data split
Disclosure of bias	Limitations of use and contraindications	Phenotypic traits such as skin tone
Risk management framework	Product integrity	Cybersecurity resilience, prevention of AI poisoning, measures for protecting user data (such as differential privacy)
Performance	Primary benefit or utility	Sensitivity, specificity, negative predictive value, positive predictive value

Labeling would directly counter the real and perceived black box problem and inform clinicians, researchers, patients, and caregivers in a manner that is equivalent to how they study, learn, and use new prescription and OTC drugs, diagnostics, and medical devices today [53,54].

New regulatory frameworks often face pushback from those they regulate, and labeling regulations are likely no exception. This resistance can be mitigated somewhat with guidance documents that incorporate feedback from various manufacturers, educational initiatives, and avenues for direct interaction between companies and regulatory bodies.

## Conclusions

AI medical products and DHTs hold immense promise, but the diverse regulatory constraints among product makers necessitate a standardized approach. This is especially critical for smaller AI developers who operate in a different landscape than health care or larger industries. To create consistency, adopting minimum product labeling requirements, understanding claims, and having substantial evidence plans become essential. As innovation accelerates, ensuring equity in the ecosystem will allow both emerging and mature technology innovators to contribute meaningfully without being hindered by not only regulatory disparities but also ambiguities, such as the uncertain classification of certain AI applications and the lack of clear communication standards. Future research exploring the various reimbursement strategies and ethical implications of AI across product makers would be valuable to providing a more complete picture of this space. The perspectives from a wide range of digital health ecosystem stakeholders should be included to ensure that their diverse needs and expectations are being addressed.

## Supplementary material

10.2196/57421 Multimedia Appendix 1 Set of linked figures referenced in Figure 1.

## Abbreviations

AI: artificial intelligence
DHT: digital health technology
FDA: Food and Drug Administration
FSIS: Food Safety and Inspection Service
FTC: Federal Trade Commission
IVD: in vitro diagnostic
OTC: over-the-counter
SaMD: software as a medical device
