# Correction: Striking a Balance: Innovation, Equity, and Consistency in AI Health Technologies

**DOI:** 10.2196/76234

**Published:** 2025-05-07

**Authors:** Eric Perakslis, Kimberly Nolen, Ethan Fricklas, Tracy Tubb

**Affiliations:** 1 Duke Clinical Research Institute, Duke University School of Medicine, 300 West Morgan Street, Durham, NC, 27701, United States, 1 9196680434; 2 Pluto Health, Durham, NC, United States; 3 Pfizer Inc, New York, NY, United States

In “Striking a Balance: Innovation, Equity, and Consistency in AI Health Technologies” (JMIR AI 2025;4:e57421) the authors noted one error.

Figure 1 originally included a citation to reference 34. This has been changed to reference 31, as pictured in the attached image.

**Figure 1.**
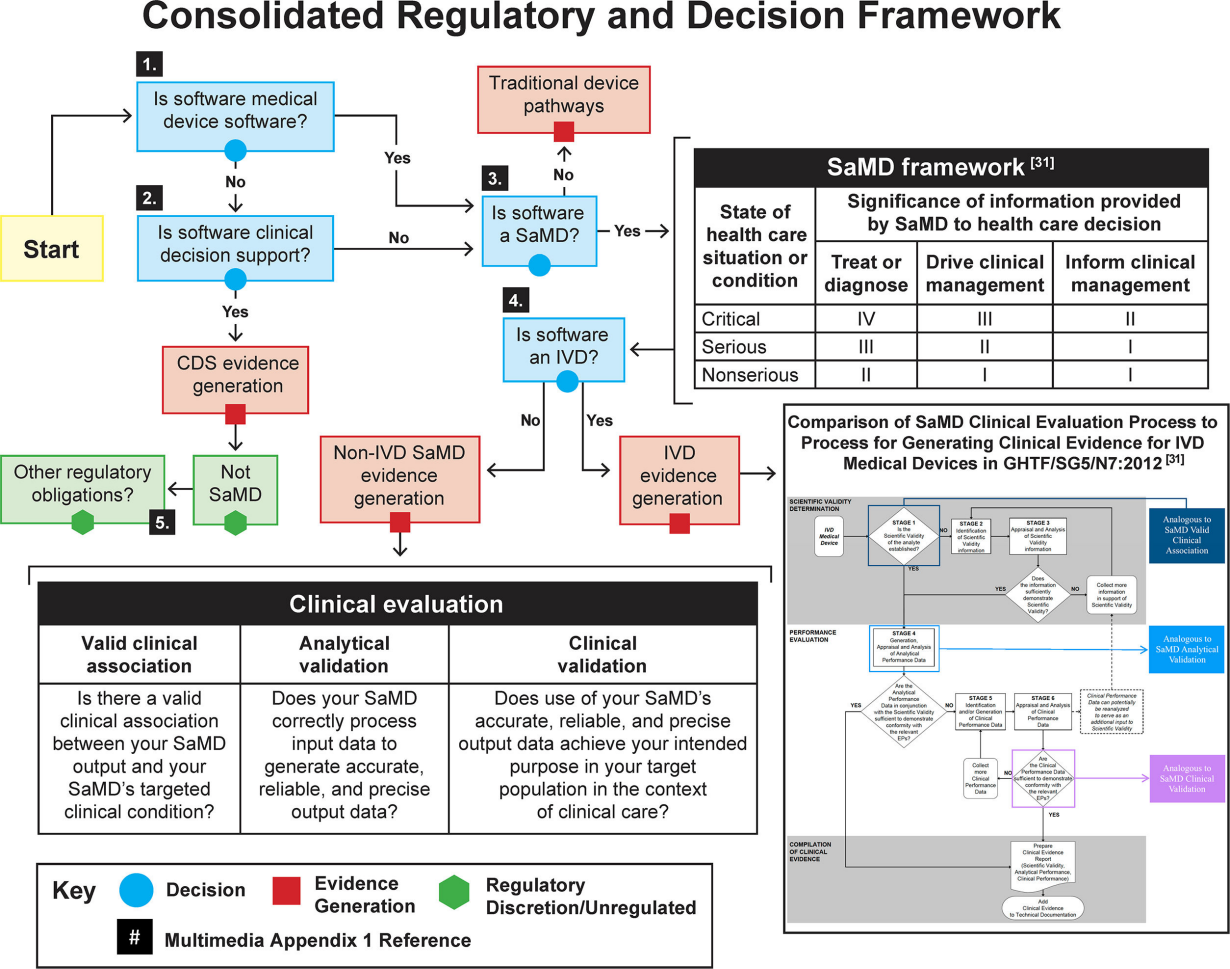
Consolidated regulatory classification decision framework (for the reader’s convenience, Multimedia Appendix 1 gives a set of figures referenced in Figure 1). CDS: clinical decision support; IVD: in vitro diagnostic; SaMD: software as a medical device.

The correction will appear in the online version of the paper on the JMIR Publications website, together with the publication of this correction notice. Because this was made after submission to PubMed, PubMed Central, and other full-text repositories, the corrected article has also been resubmitted to those repositories.

