# Supplementary figures and images for: Striking a Balance: Innovation, Equity, and Consistency in AI Health Technologies
Source: JMIR AI. 2025 Apr 7;4:e57421. doi: 10.2196/57421 (PMC12223681; doi:10.2196/57421)

## Slide 1
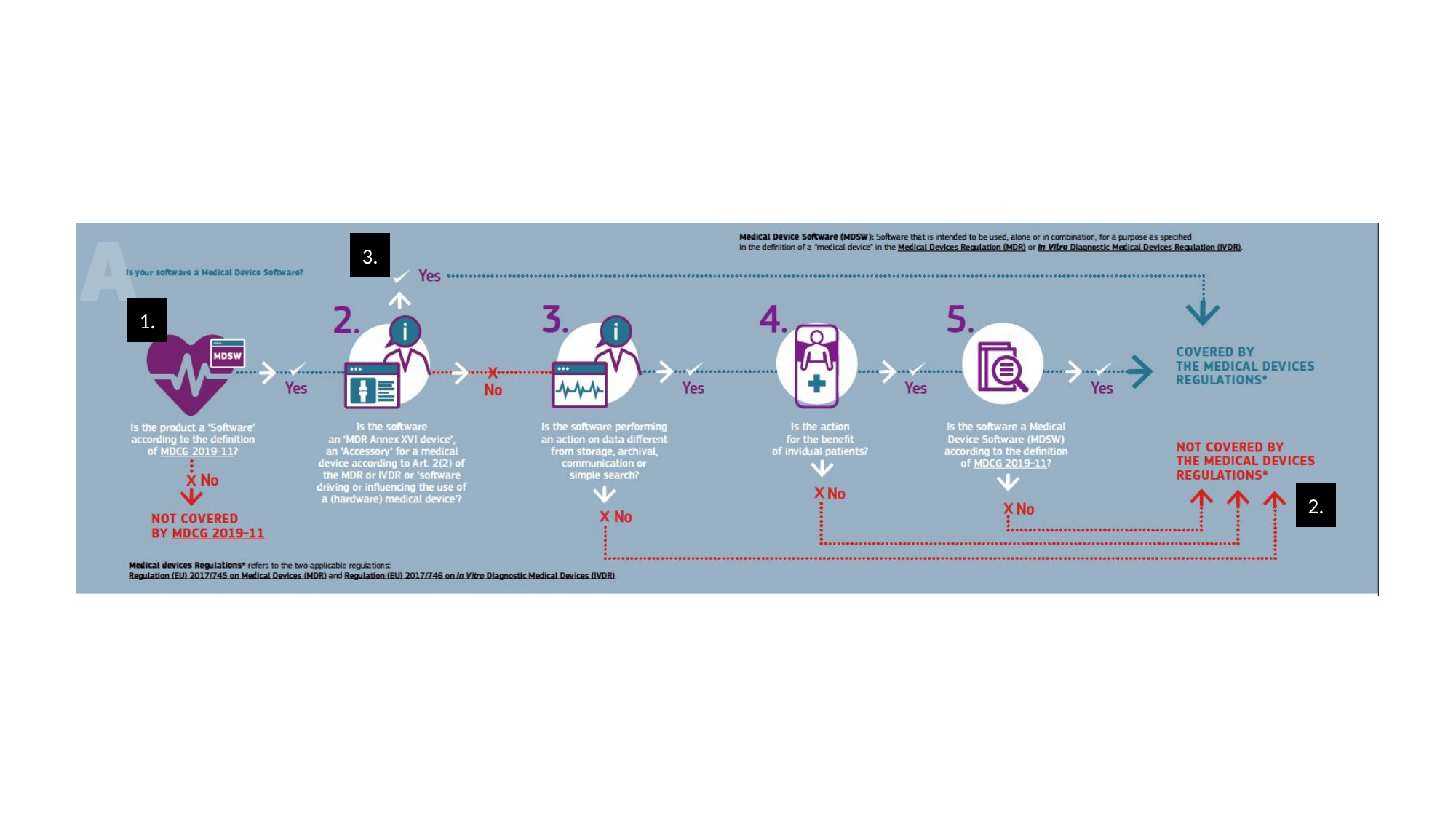

3.
1.
2.

## Slide 2
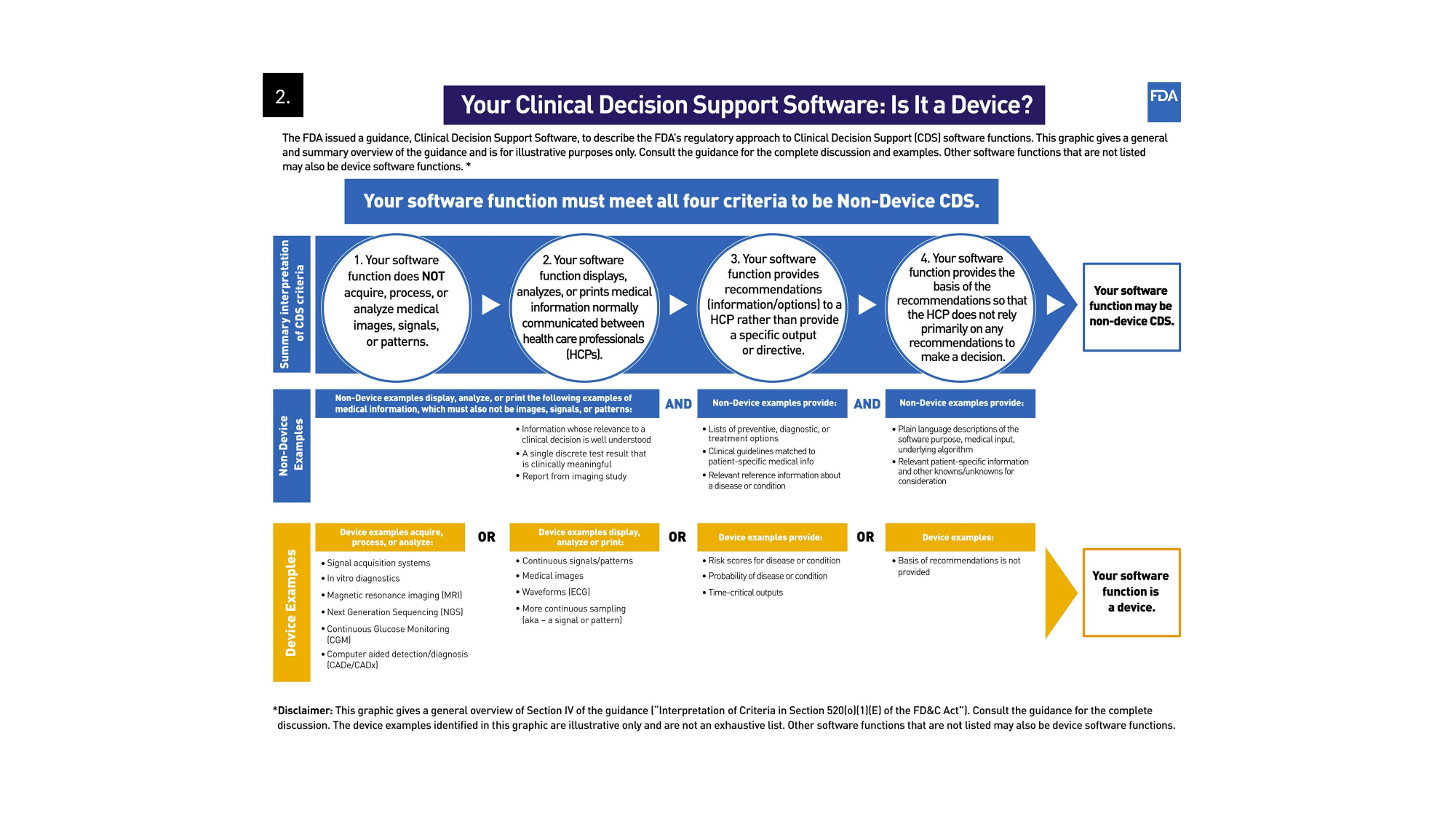

2.

## Slide 3
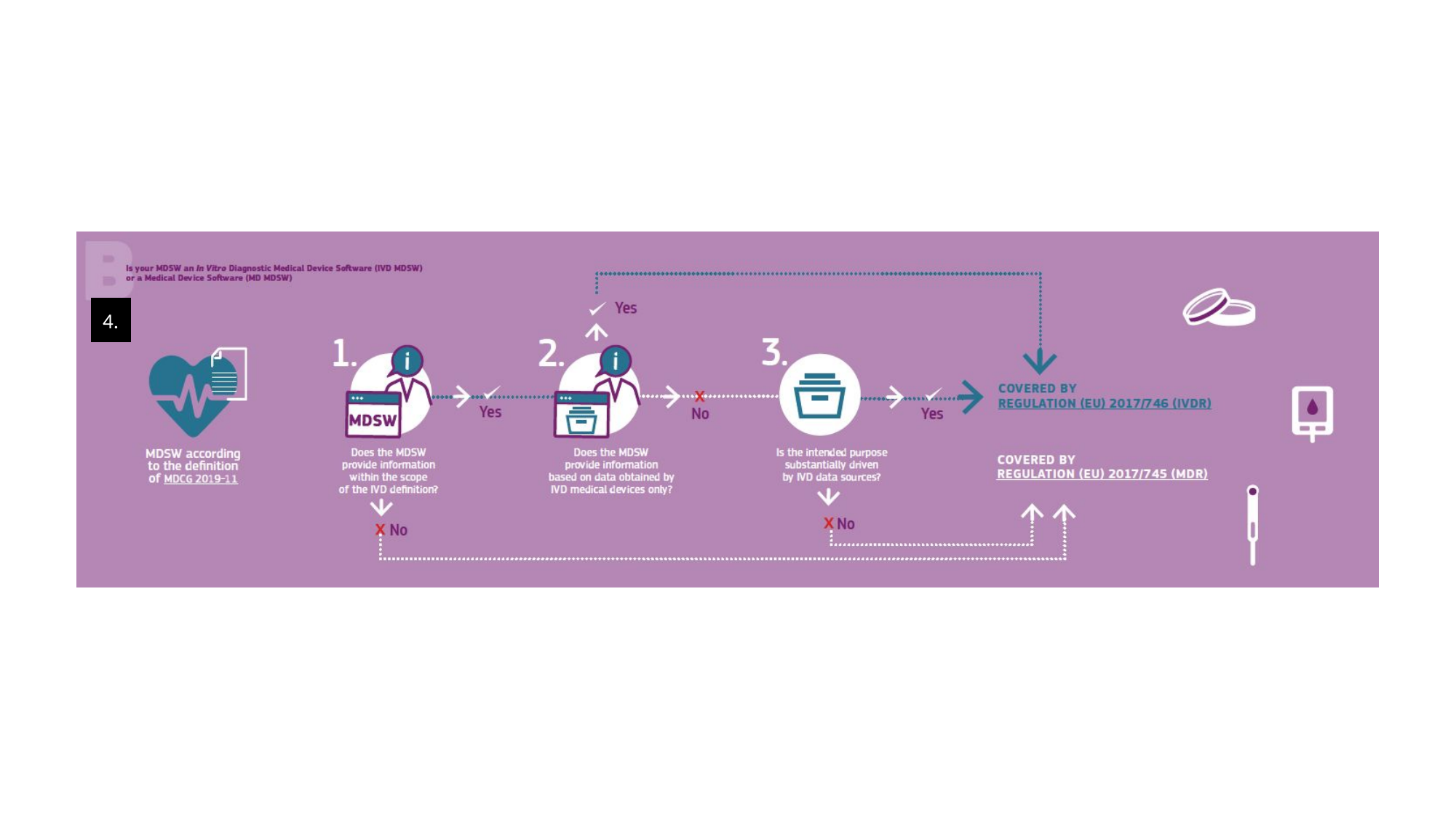

4.

Supplement: Multimedia Appendix 1 [file ai-v4-e57421-s001.pptx]
